# Four Eremophil-9-en-8-one Derivatives from *Cremanthodium stenactinium* Samples Collected in China 

**DOI:** 10.3390/molecules161210645

**Published:** 2011-12-19

**Authors:** Yoshinori Saito, Mayu Ichihara, Yasuko Okamoto, Xun Gong, Chiaki Kuroda, Motoo Tori

**Affiliations:** 1 Faculty of Pharmaceutical Sciences, Tokushima Bunri University, Yamashiro-cho, Tokushima, 770-8514, Japan; Email: tori@ph.bunri-u.ac.jp (M.T.); 2 Kunming Institute of Botany, Chinese Academy of Science, Kunming 650204, China; Email: gongxun@mail.kib.ac.cn; 3 Department of Chemistry, Rikkyo University, Nishi-Ikebukuro, Toshima-ku, Tokyo 171-8501, Japan; Email: kuroda5000144@grp.rikkyo.ne.jp

**Keywords:** Asteraceae, *Cremanthodium stenactinium*, eremophilanes, sesquiterpenes

## Abstract

Two samples of *Cremanthodium stenactinium* (Asteraceae) were collected in Sichuan Province, China. From the ethyl acetate extracts of the roots, three new eremophilane-type sesquiterpenoids and one new trinoreremophilane compound were isolated, together with other known eremophilanes. Their structures were determined based on the spectroscopic data. This is the first report of isolation of eremophilane-type compounds from the genus *Cremanthodium*.

## 1. Introduction

**Figure 1:**
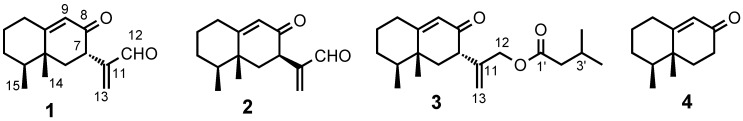
New compounds isolated in this work.

Plant species belonging to the genus *Cremanthodium* (Asteraceae) are known to grow in high mountain areas and to be very small in size [1,2]. To date there are few reports about these species, presumably due to the difficulty of collection and the scarcity of suitably sized samples. Bisabolane- and oplopane-type sesquiterpenoids and aromatic compounds were isolated from *Cremanthodium ellisii* [3,4,5,6,7], bisabolane-type and steroids from *C. discoideum* [8,9], triterpenoids from *C. potaninii* [10], and some hydrocarbons from *C. pleurocaule* [11]. However, there are no reports about the phytochemicals of *C. stenactinium*. We have been investigating both inter- and intra-specific diversity of *Ligularia* [12,13,14,15,16,17,18]. In 2009, we had an opportunity to collect two samples of *C. stenactinium* at different locations in Sichuan Province of China. From the EtOAc extracts of the root we have now isolated four new compounds, three eremophilanes **1**–**3** and trinoreremophilane **4** (Figure 1), and their structures have been determined based on the spectroscopic data. 

## 2. Results and Discussion

The molecular formula of compound **1** was determined to be C_15_H_20_O_2_ by HRMS. The IR spectrum exhibited a conjugated carbonyl group absorption at 1,695 cm^−1^. The ^13^C-NMR and HSQC spectra indicated the presence of two methyl, five methylene, four methine, and four quaternary carbon signals. The ^1^H-NMR spectrum exhibited the presence of a trisubstituted olefin and an exomethylene, as well as an aldehyde (Table 1).

**Table 1:** NMR data for compounds **1** and **2** (500 MHz for ^1^H and 125 MHz for ^13^C in C_6_D_6_).

position	^13^C (ppm)	^1^H (ppm)	
	1	1	2
1	32.7	1.72–1.81 (2H, m)	1.60–1.68 (m)
		-	1.91 (td, *J* = 12.2, 5.2 Hz)
2	26.4	1.02–1.08 (m)	0.94–1.01 (m)
		1.37–1.42 (m)	1.43–1.50 (m)
3	30.4	0.98–1.05 (m)	0.98–1.06 (m)
		1.08–1.13 (m)	1.09–1.15 (m)
4	43.5	1.00–1.05 (m)	1.50–1.55 (m)
5	39.7	-	-
6	41.9	1.67 (dd, *J *= 13.0, 5.6 Hz)	1.62 (t, *J* = 13.7 Hz)
		1.71 (dd, *J* = 13.4, 13.0 Hz)	1.73 (dd, *J* = 13.7, 4.6 Hz)
7	42.8	3.60 (dd, *J* = 13.4, 5.6 Hz)	3.64 (dd, *J* = 13.7, 4.6 Hz)
8	195.5	-	-
9	124.3	5.76 (d, *J* = 1.7 Hz)	5.80 (d, *J* = 1.3 Hz)
10	168.0	-	-
11	149.3	-	-
12	192.8	9.34 (s)	9.31 (s)
13	134.4	5.52 (s)	5.52 (s)
		5.80 (s)	5.88 (s)
14	15.5	0.71 (s)	0.63 (s)
15	15.1	0.54 (d, *J* = 6.4 Hz)	0.60 (d, *J* = 6.9 Hz)

**Figure 2:**
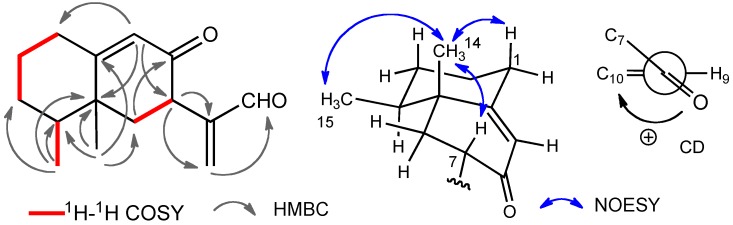
Selected 2D correlations and the sign of the CD for compound **1**.

The HMBC spectrum of compound **1** showed correlations between H-15 and C-3, C-4, and C-5, between H-14 and C-4, C-5, C-6, and C-10, between H-7 and C-11 and C-13, and between H-9 and C-1, C-5, and C-7 (Figure 2). ^1^H-^1^H COSY correlations as shown in Figure 2 were also detected. 

**Figure 3:**
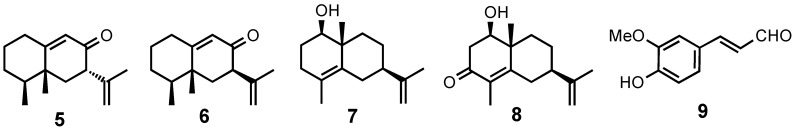
Known compounds isolated in this work.

From these results the planar structure was determined to be 8-oxoeremophila-9,11(13)-dien-12-al. The stereochemistry was revealed by the NOESY spectrum. NOEs between H-14 and H-1β, H-15, and H-7 were observed, therefore, H-7 was established to be β-oriented. The CD spectrum of compound **1** showed the Cotton effect [θ] +27000 at 237 nm (EtOH) [19] which was similar to that of the known compound, 7*S*-eremophila-9,11-dien-8-one (**5**), also found in this extract (Figure 3). The structure of **1** was established to be 4*S*,5*R*,7*S*-8-oxoeremophila-9,11(13)-dien-12-al.

The molecular formula of compound **2** was the same as that of compound **1**. The presence of a conjugated carbonyl group was also shown by the IR spectrum (1,693 cm^−1^). The ^1^H-NMR spectrum, which was very similar to that of compound **1**, exhibited the presence of an aldehyde, a trisubstituted olefin, an exomethylene, a singlet methyl, and a doublet methyl (Table 1). ^1^H-^1^H COSY correlations from H-1 through H-4 to H-15 was detected and the planar structure was determined to be as depicted in the formula (Figure 4). 

**Figure 4:**
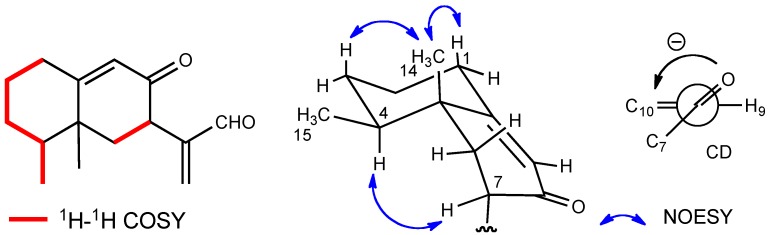
Selected 2D correlations and the sign of the CD for compound **2**.

The stereochemistry was deduced by the NOEs between H-14 and H-1β and H-3β and between H-4α and H-7α. The CD spectrum showed the Cotton effect [θ] -12000 at 225 nm (EtOH) which was similar to that of the known compound, 7*R*-eremophila-9,11-dien-8-one (**6**), also present in this extract (Figure 3). Therefore, compound **2** was established to be 4*S*,5*R*,7*R*-8-oxoeremophila-9,11(13)-dien-12-al.

Compound **3** exhibited a quasi-molecular ion peak at *m/z* 319 and the molecular formula was determined to be C_20_H_30_O_3_ by HRMS. The ^1^H-NMR spectrum indicated the presence of three doublet methyls, one singlet methyl, an exomethylene, a trisubstituted olefin, as well as two oxymethylene protons (Table 2). 

**Table 2:** NMR data for compounds **3** and **4** (500 MHz for ^1^H and 125 MHz for ^13^C; in C_6_D_6_).

position	^13^C (ppm)		^1^H (ppm)	
	3	4	3	4
1	32.6	33.1	1.74–1.83 (m)	1.76 (ddt, *J* = 14.6, 4.2, 2.0 Hz)
			-	1.83 (ddd, *J* = 14.6, 12.8, 5.2 Hz)
2	26.5	26.7	1.03–1.09 (m)	1.03–1.09 (m)
			1.38–1.43 (m)	1.39–1.44 (m)
3	30.4	30.5	1.00–1.05 (m)	0.99–1.06 (m)
			1.10–1.15 (m)	1.12–1.15 (m)
4	43.6	43.1	1.00–1.05 (m)	0.96–1.02 (m)
5	39.6	38.8	-	-
6	41.7	35.7	1.72 (dd, *J* = 12.9, 4.1 Hz)	1.27 (ddd, *J* = 14.4, 13.4, 4.4 Hz)
			1.85 (dd, *J* = 12.9, 4.1 Hz)	1.45 (ddd, *J* = 13.4, 4.9, 3.5 Hz)
7	47.5	34.3	3.12 (dd, *J* = 14.7, 4.1 Hz)	2.14 (ddd, *J* = 16.6, 14.4, 4.9 Hz)
			-	2.25 (ddd, *J* = 16.6, 4.4, 3.5 Hz)
8	196.7	197.3	-	-
9	124.4	124.7	5.73 (s)	5.80 (s)
10	168.0	168.5	-	-
11	143.9	-	-	-
12	66.3	-	4.98 (s)	-
13	114.0	-	4.98 (s)	-
			5.36 (s)	-
14	15.4	15.6	0.69 (s)	0.61 (s)
15	15.1	15.2	0.57 (d, *J* = 6.1 Hz)	0.55 (d, *J* = 6.4 Hz)
1'	172.0	-	-	-
2'	43.4	-	2.07 (d, *J* = 6.1 Hz)	-
3'	25.8	-	2.05–2.15 (m)	-
4' 5'	22.5 (2C)	-	0.84 (d, *J* = 6.4 Hz)	-

The presence of two carbonyl and two olefins was supported by the ^13^C-NMR spectrum. Therefore, this molecule should be bicyclic, because the degree of unsaturation was six and the number of the double bonds was four. The HMBC spectrum showed the correlations between H-15 and C-3, C-4, and C-5, between H-14 and C-4, C-5, C-6, and C-10, between H-7 and C-11, C-12, and C-13, between H-12 and C-1’, and other correlations shown in Figure 4. These observations indicate that this compound has an eremophilane skeleton and that 3-methylbutyryloxy group is substituted at C-12. The stereochemistry was determined by the correlations shown in Figure 4. The absolute configuration was also determined by the CD absorption of [θ] +43000 (235 nm in EtOH), which was almost the same as that of compound **1**.

**Figure 4:**
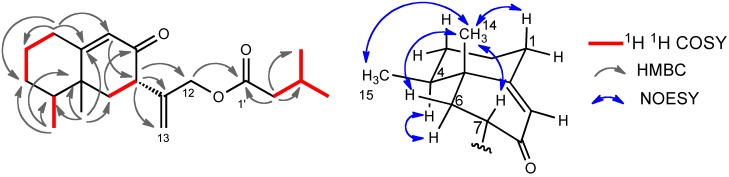
Selected 2D correlations for compound **3**.

**Figure 5:**
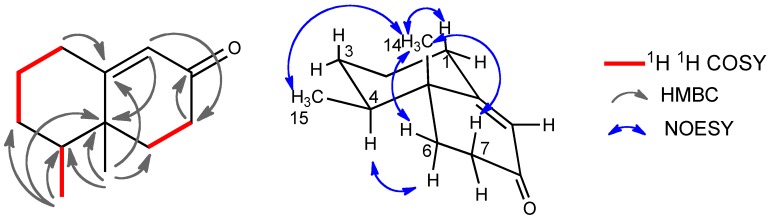
Selected 2D correlations for compound **4**.

The molecular formula of compound **4** was determined to be C_12_H_18_O by HRMS. The IR spectrum exhibited the absorption at 1,680 cm^−1^ for a conjugated ketone, which was supported by the NMR signals (δ_C_ 197.3, 168.5, 124.7; δ_H_ 5.80) (Table 2). The ^13^C-NMR spectrum showed the presence of only 12 carbon signals, including two methyl, five methylene, two methine, and three quaternary carbons. The HMBC and ^1^H-^1^H COSY correlations (Figure 5) clearly indicated the trinor-eremophilane skeleton. The NOESY spectrum showed that both methyl groups were β-oriented, and the absolute configuration was determined by the CD absorption as depicted in the formula. Compound **4** was established to be 4*S*,5*R*-trinoreremophil-9-en-8-one. This compound has been known as a synthetic intermediate as a chiral compound ([α]_D_ +185.6 (CHCl_3_)) [20], which also supported the absolute configuration of compound **4** (*vide supra*).

## 3. Experimental

### General

Specific rotations and CD spectra were measured on a JASCO P-1030 and a JASCO J-725 auto recording polarimeter; IR spectra, on a Shimadzu FT/IR-8400S spectrophotometer; ^1^H and ^13^C NMR spectra (500 MHz and 125 MHz, respectively), on a Varian 500-MR spectrometer. Mass spectra, including high-resolution ones, were recorded on a JEOL JMS-700 MStation. A Chemcopak Nucleosil 50-5 column (4.6 × 250 mm) and a hexane-ethyl acetate solvent system were used for HPLC (JASCO pump system). Silica gel BW127ZH (100–270 mesh, Fuji Silysia) was used for column chromatography. Silica gel 60 F_254_ plates (Merck) were used for TLC. 

Sample 1 (200982) was collected in the boundary between Luhuo and Seda Counties, Sichuan (N 31°42'30.2'', E 100°42'31.6''; altitude 3,700 m) and sample 2 (200938) in Litang County, Sichuan (N: 30°13'32'', E:100°16'3.6''; altitude 4,400 m) in 2009 (voucher specimens No. 200982 and 200938, were deposited in the Herbarium of Kunming Institute of Botany). Both samples were identified by X. Gong, one of the authors.

Sample 1 (200982; dried weight 21.1 g) was extracted with EtOAc to give an extract (424 mg), which was separated by column chromatography (*n*-hexane-EtOAc gradient) followed by HPLC (Nucleosil 50-5, *n*-hexane-EtOAc) to afford **1** (0.6 mg), **2** (0.3 mg), a mixture of 7*S*- and 7*R*-eremophila-9,11-dien-8-one (**5** and **6**, 60 mg), and 4-hydroxy-3-methoxycinnamaldehyde (**9**, 0.2 mg). 

Sample 2 (200938; dried weight 42.1 g) was extracted with EtOAc to give an extract (1.3 g), which was separated by column chromatography (*n*-hexane-EtOAc gradient) followed by HPLC (Nucleosil 50-5, *n*-hexane-EtOAc) to afford **3** (2.4 mg), **4** (2.1 mg), eudesma-4,11-dien-1β-ol (**7**, 1.1 mg), 1β-hydroxyeudesma-4,11-dien-3-one (**8**, 1.4 mg), 4-hydroxy-3-methoxycinnamaldehyde (**9**, 0.2 mg), and vanillin (0.2 mg) (Scheme 2). 

*4S,5R,7S-8-Oxoeremophila-9,11(13)-dien-12-al* (**1**): [α]_D_^22^ +99.5 (*c* 0.12, EtOH); FT-IR (KBr) 1695, 1676 cm^−1^; CD [θ] (EtOH) -5323 (319 nm), +27023 (237 nm); MS (CI) *m/z* 232 [M]^+^, 150, 135 (base); HRMS (CI) Obs *m/z *232.1456 (Calcd for C_15_H_20_O_2_ 232.1463).

*4S,5R,7R-8-Oxoeremophila-9,11(13)-dien-12-al* (**2**): [α]_D_^22^ −72 (*c *0.01, EtOH); FT-IR (KBr) 1693, 1679 cm^−1^; CD [θ] (EtOH) +667 (328 nm), −12025 (225 nm); MS (CI) *m/z* 233 [M+H]^+^ (base), 150, 135; HRMS (CI) Obs *m/z *233.1540 (Calcd for C_15_H_21_O_2_ 233.1541).

*4S,5R,7S-12-(3′-Methylbutyryloxy)eremophila-9,11(13)-dien-8-one* (**3**): [α]_D_^21^ +178 (*c* 0.24, EtOH); FTIR (KBr) 1737, 1677 cm^−1^; CD [θ] (EtOH) −7240 (319 nm), +43635 (235 nm); MS (CI) *m/z* 319 [M+H]^+^ (base), 217; HRMS (CI) Obs *m/z *319.2275 (Calcd for C_20_H_31_O_3_ 319.2273).

*4S,5R-Trinoreremophil-9-en-8-one* (**4**): [α]_D_^22^ +143 (*c* 0.14, EtOH); FTIR (KBr) 1680 cm^−1^; CD [θ] (EtOH) -2768 (319 nm), +34310 (238 nm); MS (CI) *m/z* 179 [M+H]^+^, 89, 61 (base); HRMS (CI) Obs *m/z *179.1422 (Calcd for C_12_H_19_O 179.1436).

## 4. Conclusions

Sample 1 afforded compounds **1** and **2**, and sample 2 compounds **3** and **4**. This is the first report of the isolation of eremophilane-type sesquiterpenoids from *Cremanthodium *spp*.* A trinoreremophilane compound, dendryphilellin A, has been isolated from the marine deuteromycete *Dendryphiella salina *[21], but there are only a few examples of simple trinoreremophilanes reported so far [22,23,24,25], which are biogenetically closely related with compounds **1**, **2**, **5**, or **6**. The present results show that eremophilane-type sesquiterpenoids are common compounds both in *Ligularia* and *Cremanthodium*, implying that the genus *Cremanthodium* is quite close to *Ligularia* or *Parasenecio* [1,2,26]. More samples are going to be investigated in the near future.
